# TWEAK/Fn14 pathway modulates properties of a human microvascular endothelial cell model of blood brain barrier

**DOI:** 10.1186/1742-2094-10-9

**Published:** 2013-01-15

**Authors:** Delphine Stephan, Oualid Sbai, Jing Wen, Pierre-Olivier Couraud, Chaim Putterman, Michel Khrestchatisky, Sophie Desplat-Jégo

**Affiliations:** 1 Aix-Marseille Université, NICN, UMR7259, 13344, Marseille, France; 2 NICN, UMR 7259, Faculté de Médecine Nord, Bd Pierre Dramard, CS80011, 13344, Marseille Cedex 15, France; 3 Albert Einstein College of Medicine, New York, USA; 4 Institut Cochin, CNRS UMR 8104-INSERM U567, Université René Descartes, Paris, France

**Keywords:** CCL-2, hCMEC/D3, HMEC, IL-8, MMP-9, Neuroinflammation, TNFSF12, ZO-1

## Abstract

**Background:**

The TNF ligand family member TWEAK exists as membrane and soluble forms and is involved in the regulation of various human inflammatory pathologies, through binding to its main receptor, Fn14. We have shown that the soluble form of TWEAK has a pro-neuroinflammatory effect in an animal model of multiple sclerosis and we further demonstrated that blocking TWEAK activity during the recruitment phase of immune cells across the blood brain barrier (BBB) was protective in this model. It is now well established that endothelial cells in the periphery and astrocytes in the central nervous system (CNS) are targets of TWEAK. Moreover, it has been shown by others that, when injected into mice brains, TWEAK disrupts the architecture of the BBB and induces expression of matrix metalloproteinase-9 (MMP-9) in the brain. Nevertheless, the mechanisms involved in such conditions are complex and remain to be explored, especially because there is a lack of data concerning the TWEAK/Fn14 pathway in microvascular cerebral endothelial cells.

**Methods:**

In this study, we used human cerebral microvascular endothelial cell (HCMEC) cultures as an *in vitro* model of the BBB to study the effects of soluble TWEAK on the properties and the integrity of the BBB model.

**Results:**

We showed that soluble TWEAK induces an inflammatory profile on HCMECs, especially by promoting secretion of cytokines, by modulating production and activation of MMP-9, and by expression of cell adhesion molecules. We also demonstrated that these effects of TWEAK are associated with increased permeability of the HCMEC monolayer in the *in vitro* BBB model.

**Conclusions:**

Taken together, the data suggest a role for soluble TWEAK in BBB inflammation and in the promotion of BBB interactions with immune cells. These results support the contention that the TWEAK/Fn14 pathway could contribute at least to the endothelial steps of neuroinflammation.

## Background

TWEAK (tumor necrosis factor-like weak inducer of apoptosis) is a member of the TNF ligand family and has been described in both membrane and soluble forms [1]. It is now admitted that TWEAK is involved in the regulation of many human pathologies, including lupus nephritis, rheumatoid arthritis, and inflammatory bowel diseases [2-5]. Moreover, increasing amounts of data support the contention that TWEAK may play a dual role in physiological versus pathological tissue responses (for a review see [6]). By binding to its main receptor, Fn14, TWEAK is known to induce proliferation of endothelial cells *in vitro* and angiogenesis *in vivo*[7,8]. Using transgenic mice that overexpress soluble TWEAK, we have shown that the soluble form of TWEAK has a pro-neuroinflammatory effect in an animal model of multiple sclerosis (MS) [9]. Subsequently, using anti-TWEAK monoclonal antibody injections in this model followed by histopathological studies, we demonstrated that blocking TWEAK activity during the recruitment of immune cells across the blood brain barrier (BBB) was protective [10]. The BBB constitutes a physical and metabolic barrier that separates the CNS from the circulatory system. It is composed of specialized brain microvascular endothelial cells in close interaction with pericytes and astrocytic end feet, and bound together by tight junctions. Tight junctions between endothelial cells are formed by transcellular proteins, including claudins and occludin, that interact with the cytoskeleton via cytoplasmic proteins, such as zonula occludens-1 (ZO-1).

Increased permeability of the BBB is an early and critical event in the development and evolution of brain inflammatory diseases. It is now well established that endothelial cells and astrocytes, two of the major cellular components of the BBB, are targets of TWEAK [7,11]. Moreover, it has been shown that, when injected into mice brains, TWEAK disrupts the architecture of the BBB and induces expression of matrix metalloproteinase-9 (MMP-9) in the brain [12]. MMPs constitute a family of zinc-dependent secreted or cell surface-associated endopeptidases that cleave matrix components and a variety of pericellular proteins, including cytokines, cell surface receptors, and adhesion molecules. MMP-2 and MMP-9 (also known as gelatinases) are probably among the most studied of the MMPs in the CNS and intravenous administration of MMP-9 *in vivo* has been shown to alter the properties of the BBB [13-16].

The importance of TWEAK in brain pathology is further evidenced by data proving that TWEAK blocking antibodies or Fn14 decoy receptors are efficient in animal models of ischemic stroke and brain edema [17-19]. Nevertheless, the mechanisms involved are complex and, at times, results appear paradoxical; for instance, treatment with TWEAK renders neurons tolerant to a lethal hypoxic or ischemic injury [20]. A recent study on post-mortem brain tissue from patients with MS indicates that TWEAK is increased in meningeal macrophages, in astrocytes, and in microglia associated with lesions and vascular abnormalities, and that Fn14 is mainly localized in neurons and reactive astrocytes of the cerebral cortex in highly infiltrated MS brains [21]. Interestingly, we have shown that in MS patients, monocytes but not lymphocytes express membrane TWEAK [22]. Taken together, the published data suggest a role for membrane or soluble TWEAK in promoting monocyte interaction with the BBB, BBB inflammation, or monocyte diapedesis, and support the contention that the TWEAK/Fn14 pathway could at least contribute to the endothelial steps of neuroinflammation. However, the molecular mechanisms involved in the effects of TWEAK on the BBB remain to be determined. In this study, we formed an *in vitro* model of the BBB using human cerebral microvascular endothelial cell (HCMEC) cultures to study the effects of soluble TWEAK on the properties and integrity of the BBB. We showed that soluble TWEAK induces an inflammatory profile on HCMEC, especially by promoting secretion of cytokines, by modulating production and activation of MMP-9, and expression of cell adhesion molecules. We also demonstrated that these effects of TWEAK are associated with increased permeability of the HCMEC monolayer in the *in vitro* BBB model.

## Methods

### Cells and culture reagents

The human brain endothelial cell line hCMEC/D3 is described in [23]. hCMEC/D3 cells were seeded on Transwell® filters (polycarbonate 12 well, pore size 3.0 μm, Corning, Lowell, MA) coated with type I collagen (BD Biosciences, Paris, France), at a density of 350,000 cells/cm^2^ in commercially available complete medium EGM®-2 (Lonza, Walkersville, MD), supplemented with vascular endothelial growth factor, insulin-like growth factor 1, epidermal growth factor, basic fibroblast growth factor (FGF), hydrocortisone, ascorbate, penicillin-streptomycin, and 2.5% FCS, (all from Lonza) in an incubator at 37°C with 5% CO_2_. For differentiation and expression of junction-related proteins, the hCMEC/D3 cells were grown at confluence in a growth-factor-depleted medium.

Primary HCMECs (Cell Systems, Kirkland, WA) were grown on 0.2% gelatin-coated (Fisher Scientific, New York, NY) tissue-culture plates in M199 medium supplemented with 20% fetal bovine serum (Gibco/BRL, Grand Island, NY), 5% heat-inactivated human serum (Invitrogen, Carlsbad, CA), 1% penicillin-streptomycin (Gibco/BRL), and 12 ng/ml endothelial cell growth factor (Sigma Aldrich, St. Louis, MO).

Human umbilical vein endothelial cells (HUVECs) and a human acute monocytic leukemia cell line (THP-1) were obtained from ATCC (Molsheim, France) and were cultivated, respectively, in EBM-2 medium supplemented with EBM-2 bullet kit (Lonza) and RPMI 1640 supplemented with 10% FCS and 1% penicillin-streptomycin (Invitrogen).

For stimulation assays, cells were incubated for 3 h, 12 h, or 24 h with recombinant human TWEAK (100 ng/ml, Peprotech, Neuilly-Sur-Seine, France), Fc-TWEAK (1 μg/ml, BiogenIdec, Cambridge, MA), its isotype control P1.17 (BiogenIdec), or recombinant human TNF (10 ng/ml, Peprotech). In some experiments, cells were incubated with recombinant human MMP-9 (rhMMP-9) from Calbiochem. All reagents were endotoxin-free.

### Flow cytometry

After trypsination, differentiated unstimulated or TWEAK-stimulated hCMEC/D3 cells were pre-incubated on ice for 20 min with a solution containing PBS, 1% FCS, 0.02% sodium azide, and 25% purified human serum Immunoglobulin G(Sigma Aldrich) to inhibit binding to Fc receptors. After washes with a solution containing PBS, 1% FCS, and 0.02% sodium azide, cells were incubated on ice for 20 min with fluorescein-conjugated anti-human ICAM-1 antibody, fluorescein-conjugated anti-human E-selectin antibody (both from R&D Systems, Minneapolis, USA), or anti-human Fn14 phycoerythrin-conjugated antibody (eBioscience, Paris, France). After three more washes, cells were centrifuged and resuspended in PBS with 2% paraformaldehyde. Fluorescence-activated cell sorting (FACS) analysis was performed on a FACSCanto II (Becton Dickinson, Le Pont-De-Claix, France) using BD FACSDiva software.

### RNA extraction and RT-PCR analysis

Total RNA was prepared from cultures of hCMEC/D3, HUVECs, and THP-1 using the RNeasy Lipid Tissue Mini kit (Qiagen, Courtaboeuf, France). Single-strand cDNA was synthesized from 1 μg of total RNA using oligo(dT)_12–18_ primers (Invitrogen) and Moloney murine leukemia virus reverse transcriptase (Invitrogen) under the conditions indicated by the manufacturer. The sequences of the specific forward (F) and reverse (R) primers were as follows: TWEAK-F (5′-ATATATAGATCTATGGCCGCCCGTCGGAGC-3′), TWEAK-R (5′-AGCCTTCCCCTCATCAAAGT-3′), Fn14-F (5′-CCAAGCTCCTCCAACCACAA-3′), Fn14-R (5′-TGGGGCCTAGTGTCAAGTCT-3′), GAPDH-F (5′-GTCAGTGGTGGACCTGACCT-3′), and GAPDH-R (5′-TGCTGTAGCCAAATTCGTT-3′). Each cDNA was amplified by *Taq* recombinant DNA polymerase (Invitrogen). The cDNAs were first denatured 3 min at 94°C, then 40 PCR cycles were carried out with the following profile: 45 s denaturation at 94°C, 30 s annealing at 59°C for Fn14 or 52°C for GAPDH, and 1 min elongation at 72°C. Cycles were followed by a 10 min final elongation at 72°C (Verity 96-well thermal cycler, Applied Biosystems Foster City, CA). Controls were performed with template-free PCR reactions. PCR products were analyzed by electrophoresis on a 2% agarose gel containing ethidium bromide. The expected sizes of the TWEAK, Fn14, and GAPDH PCR products were 522 base pairs (bp), 242 bp, and 226 bp, respectively.

### TaqMan quantitative PCR

Real-time PCR (qPCR) experiments were carried out with the 7500 Fast Real-Time PCR System (Applied Biosystems). All reactions were performed using TaqMan Fast Universal PCR Master Mix and two probes from the TaqMan® Gene Expression Assays (MMP-9, Hs00234579_m1 and Abelson (ABL): ABL-F (5′-TGGAGATAACACTCTAAGCATAACTAAAGGT-3′), ABL- TaqMan reverse probe (5′-Fam6CCATTTTTGGTTTGGGCTTCACACCATT-Tamra-3′), ABL-R (5′-GATGTAGTTGCTTGGGACCCA-3′, used as reference) according to the manufacturer’s instructions (Applied Biosystems). Each experiment used 25 ng of previously prepared hCMEC/D3 cDNA. Samples were run in duplicates on the same 96-well plates and analyzed with the 7500 Software v2.0 (Applied Biosystems). The thermal cycling conditions started with initial denaturation at 95°C for 20 s, followed by 40 cycles of denaturation at 95°C for 3 s and annealing and extension at 60°C for 30 s. Relative expression levels are determined according to the ΔΔCt method where the expression level of the mRNA of interest is given by 2^-ΔΔCT^ where ΔΔCT = ΔCT target mRNA − ΔCT reference mRNA (*Abelson*) in the same sample.

### Bromodeoxyuridine assay

hCMEC/D3 cell proliferation was determined by measurement of bromodeoxyuridine (BrdU) incorporation during DNA synthesis by chemiluminescence detection using the Cell Proliferation ELISA BrdU kit (Roche Applied Science, Meylan, France) according to the manufacturer’s instructions.

### Cytokine production

hCMEC/D3 differentiated cells were stimulated with TWEAK or TNF for 24 h. Supernatants were collected, centrifuged, and stored at −80°C until analysis. CCL-2, IL-8, Il-6, and IL-10 levels were evaluated using commercially available ELISA kits (Peprotech, and BD Biosciences, San Jose, CA) according to the manufacturers’ instructions. All samples were analyzed in triplicate. The detection threshold was 16 pg/ml of cytokine.

### Transport assay

For transport experiments, we tested the passage of two distinct molecules, Lucifer yellow (LY) (Sigma) and BSA-FITC (Accurate Chemical and Scientific, Westbury, NY). hCMEC/D3 cells or HCMECs were seeded and differentiated on coated Transwell® filters. Both the upper and lower chambers were washed with pre-warmed Ringer-HEPES (RH) at 37°C. At time t = 0, LY or BSA-FITC was applied in the apical compartment. After 60 min, detection of the fluorescent molecules was carried out with a Beckman DTX800 luminometer with excitation at 430/485 nm, and emission at 535 nm. Permeability coefficients (Pe) take into account the relation between the permeability of the monolayer and the permeability of empty filters (pre-coated, without cells). Each condition was tested in triplicate in each experiment.

### Transepithelial electric resistance measurements

The assay setup of HCMECs for transepithelial electric resistance (TEER) was the same as for the BBB permeability assay. The HCMEC TEER was measured using the Electrical Resistance System (Millicell-ERS-2; Millipore, Bedford, MA), following the manufacturer’s instructions. In brief, coated inserts without cells were used as a blank (minimum resistance). The electrical resistance of each insert following treatment with TWEAK, P1.17, or TNF was calculated by subtracting the blank from each reading. Each condition was run in duplicate, and the resistance measured twice for each well.

### Western blot analysis

The following antibodies were used: goat anti-TWEAK (R&D system, Abingdon, UK), rabbit anti-ZO-1 (1/200, Invitrogen), and mouse anti-β-actin (1/3000, Sigma Aldrich). Protein concentrations were determined using the Lowry method (Bio-Rad, Hercules, CA). After boiling, aliquots containing equal amounts of protein were loaded in Laemmli buffer and separated by 8.5% sodium dodecyl sulphate polyacrylamide gel electrophoresis (SDS PAGE, Bio-Rad) using a MiniBlot system (Bio-Rad). Proteins were transferred onto nitrocellulose membranes (Amersham Biosciences, Buckinghamshire, UK) in transfer buffer (25 mM Tris, 192 mM glycine, and 20% ethanol). Membranes were incubated overnight in blocking buffer at 4°C and then probed with the primary antibody against ZO-1, β-actin, or TWEAK diluted in blocking buffer (Roche Diagnostics, Mannheim, Germany). After washing, membranes were incubated with a peroxidase-conjugated secondary antibody (Jackson Immunoresearch, West Grove, PA). Finally, proteins were detected using a chemiluminescence kit (Roche Diagnostics). Films were digitized using GeneTools software (Syngen, San Carlos, CA), and optical densities of the bands were assessed using Scion Image software (Scion Corporation, MA).

### *In situ* zymography

To assess gelatinolytic activity of MMPs, hCMEC/D3 cells were grown on glass coverslips and the medium was supplemented to a final concentration of 5 mM CaCl_2_ and 10 μg/ml of intramolecularly quenched FITC-labeled DQTM-gelatin (EnzCheck Collagenase kit from Molecular Probes), as previously described [24,25]. After 2 h at 37°C in a humidified atmosphere containing 5% CO_2_, cells were rinsed in PBS, fixed with 4% paraformaldehyde (PFA) for 5 min, and incubated for 5 min with DNA intercalant Hoechst #33258 (Molecular Probes, Eugene, OR). Cells were observed with a Nikon E800 upright epifluorescence microscope and digital images were acquired at 1,024 × 1,024 pixels and saved in TIFF format. Fluorescence levels were measured at the level of individual cells using image J software; image editing was performed using Adobe Photoshop (Adobe Systems, Paris, France).

### Gel zymography

Standard methodology for gelatin zymography was used to detect MMP-2 and MMP-9 expression levels via their activity in cell supernatant or cell lysate samples, as described previously [25]. Serum-free culture supernatants and lysates were collected and protein concentration was normalized as mentioned above. Equal amounts of protein were subjected to 8% SDS PAGE containing 1 mg/ml gelatin (Sigma Aldrich) in nondenaturing, nonreducing conditions. After electrophoresis, gels were washed twice for 30 min in 2.5% Triton X-100 to remove SDS and incubated for 48h in MMP-activating buffer, 50 mM Tris–HCl, pH 7.5, with 10 mM CaCl_2_ at 37°C. Gels were then stained with 0.1% Coomassie Brilliant Blue R-250 (Bio-Rad) for 3h and destained with a solution containing 5% acetic acid until clear bands of gelatinolysis appeared on a dark background. Gels were digitized using GeneTools software.

### Immunohistochemistry

hCMEC/D3 cells grown on glass coverslips were fixed in 4% PFA and were incubated for 1 h at room temperature with rabbit anti-ZO-1 (Invitrogen) or goat anti-MMP-9 (Abcam, Cambridge Science Park, UK) primary polyclonal antibodies. Subsequently, cells were incubated with Alexa Fluor 488 or 594 anti-mouse or anti-goat secondary antibodies (Invitrogen) followed by Hoechst and mounted in fluorescent mounting medium (DAKO, Glostrup, Denmark). The mounted slides were observed with a Leica TCS SP2 confocal microscope (Leica Microsystems, Heidelberg, Germany). High magnification images were acquired using a 633 HCX PL APO oil immersion objective by sequential scanning to minimize the crosstalk of fluorophores. For each channel, photomultiplier gains and offsets were adjusted to use full image dynamic range. Images acquired at 1,024 × 1,024 pixels and saved in TIFF format were processed for colocalization analysis using ImageJ plug-in processing software. Image editing was performed using Adobe Photoshop.

### MAPK inhibition studies

To investigate the involvement of mitogen-activated protein kinases (MAPKs) in MMP-9 expression following TWEAK stimulation, we blocked c-RAF1 and MEK signaling pathways using ERK2 and MEK1/2 specific inhibitors (Sigma Aldrich) at working concentrations of 5 μM GW5074 and 0.5 μM U0126v. hCMEC/D3 cells were cultured for 24h in the presence or absence of TWEAK (100 ng/ml) and in the presence or absence of the MAPK inhibitors; cell lysates and supernatants were then collected and tested by gel zymography.

## Results

### hCMEC express Fn14 and are a target of TWEAK

**Figure 1:**
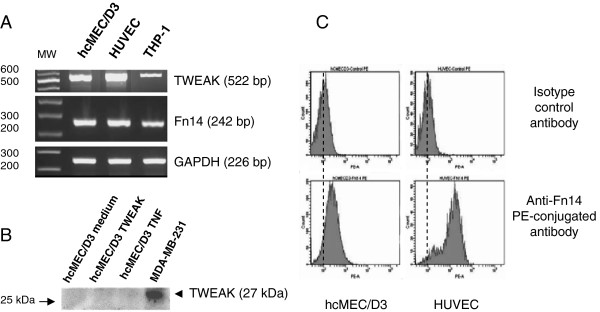
**Expression of TWEAK and its receptor, Fn 14 by hCMEC/D3 cells.** (**A**) Steady-state levels of TWEAK, Fn14, and GAPDH mRNAs were assessed by semi-quantitative RT-PCR in cultures of hCMEC/D3, HUVECs, and THP-1 cells. PCR products were analyzed by electrophoresis on a 2% agarose gel containing ethidium bromide and PCR products of the expected sizes were obtained for TWEAK (522 bp), Fn14 (242 bp), and GAPDH (226 bp) indicating expression of all three genes at the mRNA level. (**B**) Western blot analysis of protein extracts from hCMEC/D3 or MDA-MB-231 cells (positive control) following separation by SDS PAGE and transfer onto nitrocellulose membranes. Membranes were probed with a primary antibody against TWEAK. Note the absence of TWEAK protein expression by the hCMEC/D3 cells as compared with the MDA-MB-231 cells. (**C**) FACS analysis of membrane bound Fn14 on differentiated hCMEC/D3 and HUVECs using anti-human Fn14 or isotype control phycoerythrin-conjugated antibodies. Note the presence of Fn14 at the plasma membrane of the hCMEC/D3 cells.

In a first step, we used RT-PCR to study, in the hCMEC/D3 cells, the expression of the mRNAs encoding TWEAK and its receptor Fn14. We found that hCMEC/D3 cells express both TWEAK and Fn14 mRNAs (Figure 1A). We next used flow cytometry to assess TWEAK and Fn14 expression at the membrane in the same cells. We show that hCMEC/D3 cells do not constitutively express membrane TWEAK but constitutively express Fn14 on their surface (Figure 1C). TWEAK exposure did not up-regulate TWEAK or Fn14 expression at the cell surface (data not shown). Similarly, using ELISA, we were not able to detect soluble TWEAK in the culture supernatants of hCMEC/D3 (data not shown). In agreement with data issued from Western blot analysis of TWEAK expression by hCMEC/D3 cells (Figure 1B), these results lead us to conclude that this cell line expresses neither membrane nor soluble TWEAK.

### TWEAK induces inflammation of HCMEC

**Figure 2:**
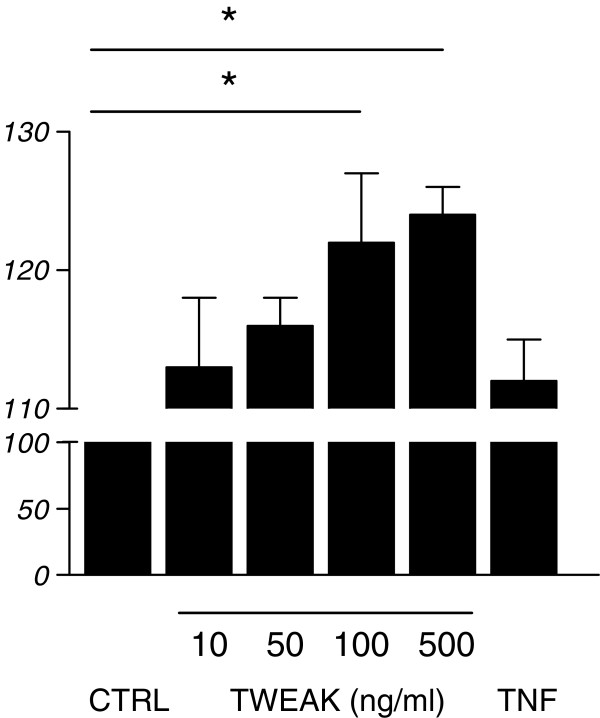
**Proliferative effects of TWEAK on hCMEC/D3.** Unstimulated (CTRL) or stimulated (TWEAK, TNF) hCMEC/D3 cell proliferation was determined by measurement of BrdU incorporation during DNA synthesis by chemiluminescence detection. Results indicate increased hCMEC/D3 cell proliferation on TNF and TWEAK stimulation, the latter being more prominent. * *p* < 0.05 according to Student’s *t* test.

Next, we studied the effects of TWEAK on hCMEC/D3 proliferation using a BrdU incorporation test. As indicated in Figure 2, a 24h TWEAK exposure of the cells induced proliferation. It is worth noting that the proliferative effects of soluble TWEAK are significantly higher than those of TNF.

**Figure 3:**
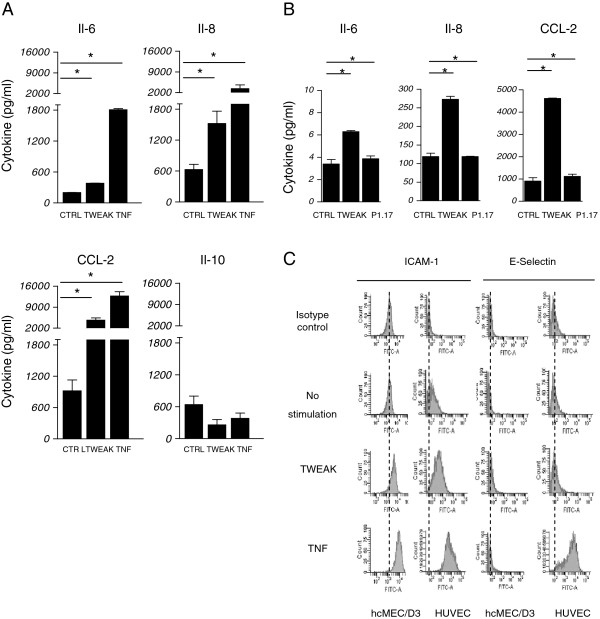
**hCMEC/D3 cells produced chemokines and membrane ICAM-1 after TWEAK exposure.** (**A**) ELISA analysis of CCL-2, IL-8, Il-6, and IL-10 levels in the supernatants of hCMEC/D3 differentiated cells stimulated with TWEAK or TNF for 24 h or not (CTRL); all samples were analyzed in triplicates. The detection threshold was 16 pg/ml of cytokine. Note the increased secretion of CCL-2, Il-8, and Il-6*.* (**B**) Primary HCMECs were stimulated with Fc-TWEAK or its isotype control P1.17 for 24 h or not (CTRL). Supernatants were collected and Il-6, IL-8, and CCL-2; levels were evaluated by ELISA. All samples were analyzed in triplicate. The detection threshold was 16 pg/ml of cytokine. (**C**) FACS analysis of membrane expression of ICAM-1 and E-selectin in differentiated hCMEC/D3 and HUVEC cells stimulated with TWEAK or TNF for 24 h or not (CTRL). Cells were incubated with anti-human ICAM-1 and E-selectin or isotype control fluorescein-conjugated antibodies. TWEAK induces ICAM-1 labeling at the membrane of hCMEC/D3 cells. In (**A**) and (**B**), * *p* < 0.05 according to Student’s *t* test.

To assess the potential inflammatory effects of TWEAK on hCMEC/D3 cells and BBB inflammation, we used ELISA to measure cytokine secretion in the culture supernatants after TWEAK or TNF exposure during 24h, compared with nonstimulated cells (Figure 3). We found that TWEAK significantly up-regulated proinflammatory cytokine (CCL-2, Il-8, and Il-6) production in the hCMEC/D3 cells (Figure 3A). We obtained the same results with primary HCMECs (Figure 3B). We also showed that nonstimulated cells can produce low levels of the anti-inflammatory cytokine Il-10 and that this secretion is not significantly modulated by TWEAK or TNF.

We also studied the effects of TWEAK stimulation on the expression of hCMEC/D3 adhesion molecules. We chose to explore E-selectin, which is involved in leukocyte rolling, and ICAM-1, which is also up-regulated during inflammation, especially during firm leukocyte adhesion to the endothelium. Using flow cytometry, we showed in hCMEC/D3 that E-selectin membrane expression is not significantly up-regulated by TWEAK, while ICAM-1 membrane levels are clearly increased following a 24h TWEAK exposure (Figure 3C).

### TWEAK increases leakiness of the *in vitro* model of the BBB

**Figure 4:**
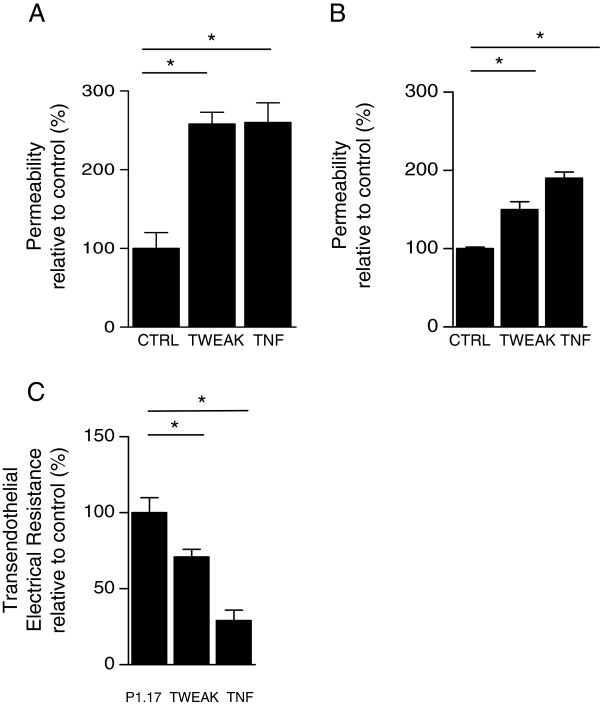
**WEAK increased Lucifer yellow permeability in an *in vitro *model of the BBB.** (**A**) hCMEC/D3 cells were differentiated on coated Transwell® filters and stimulated (TWEAK, TNF) or not (CTRL) during 24 hours. At time t = 0, Lucifer yellow (LY) was applied in the apical compartment. After 60 min, LY fluorescence was assessed in the lower compartment and the permeability coefficient (Pe) was calculated taking into account the relation between the permeability of the monolayer and the permeability of empty filters (pre-coated, without cells). Each condition was run in triplicate in three independent experiments. TWEAK induces increased passage of LY across the hCMEC/D3 cell monolayer. (**B**) The passage of BSA-FITC was used to assess transport through primary HCMEC seeded and differentiated on coated filters. Primary HCMEC were stimulated with Fc-TWEAK or TNF for 24 h or not (CTRL). At time t = 0, BSA-FITC was applied in the apical compartment. After 60 min, the fluorescence levels were assessed in the basal compartment. Each condition was run in triplicate. (**C**) The assay setup of HCMEC for TEER was the same as for the BBB permeability assay described in (**B**). HCMEC TEER was measured by the Electrical Resistance System Millicell-ERS-2. Coated inserts without cells were used as a blank (minimum resistance). The electrical resistance of each insert following treatment with TWEAK, P1.17, or TNF was calculated by subtracting the blank from each reading. Each condition was run in duplicate, and the resistance measured twice for each well. In (**A**), (**B**) and (**C**) * *p* < 0.05 according to Student’s *t* test.

Lucifer yellow is a small hydrophobic molecule that presents low cerebral penetration and that is classically used as a molecular marker of paracellular passage. To determine the effects of TWEAK on the permeability of a monolayer of brain endothelial cells, hCMEC/D3 cells were grown on Transwell filters and were exposed for 24h to TWEAK or TNF. We observed that TWEAK induced a significant increase in Pe compared with the nonstimulated condition Figure 4A. Similar results were obtained with human primary HCMEC (Figure 4B). In addition, we evaluated the TEER of the *in vitro* BBB model upon TWEAK or TNF stimulation; we observed decreased TEER upon TWEAK stimulation (Figure 4C). These data, associated with results from transport experiments, lead us to conclude that soluble TWEAK increases permeability of the human monolayers of brain endothelial cells in the *in vitro* BBB model.

### TWEAK stimulates MMP-9 expression and activity via MAPK signaling pathway

**Figure 5:**
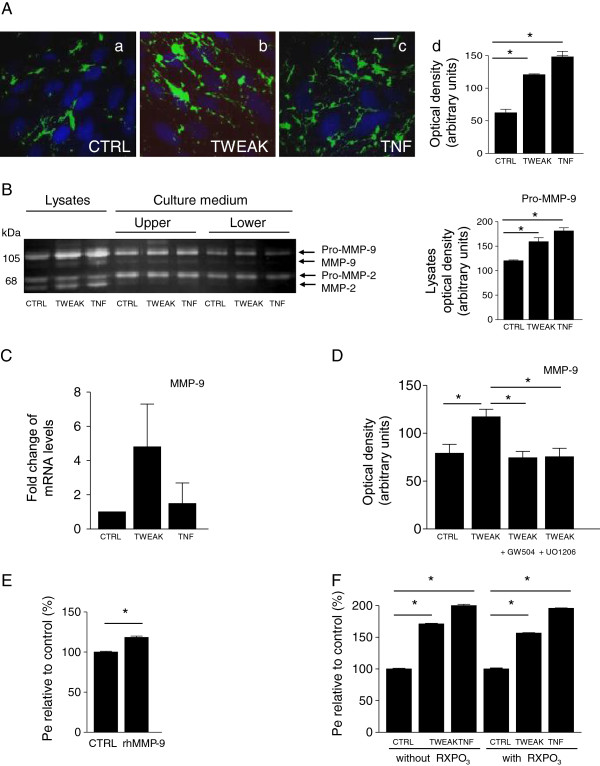
**Gelatinase activity and expression in hCMEC/D3 cells treated with TWEAK and effects of MMP inhibition.** (**A**) Epifluorescence photomicrographs in live hCMEC/D3 cells with gelatin-quenched fluorescent substrate (green), and nuclear intercalant Hoechst (blue). Net gelatinolytic activity (**a**) increased with TWEAK (**b**) and TNF (**c**), shown by densitometric analysis (**d**). Scale bar, 10 μm. (**B**) Gel zymography showing expression and secretion of MMP-2 and MMP-9 in cell lysates and culture media after treatment of hCMEC/D3 with TWEAK or TNF. Nonstimulated endothelial cells express pro-MMP-9, pro-MMP-2, and active MMP-2. Active MMP-9 is barely detected. Control cells secrete pro-MMP-9 and pro-MMP-2 in both compartments, and low levels of their active forms. In cell lysates, TWEAK or TNF treatment has no significant effect on the expression of pro- and active MMP-2 but significantly induces pro-MMP-9 and active MMP-9 expression; there is no effect on the secreted forms of MMP-2 and MMP-9. (Densitometric analysis of pro-MMP-9 zymograms of the lysates.) (**C**) MMP-9 mRNA quantification by qPCR, expressed as fold change ratios of treated versus control samples after normalization with Abelsson mRNA. (**D**) Inhibition of TWEAK-induced MMP-9 activity by ERK and JUNK inhibitors in hCMEC/D3 cells. After serum-starvation for 24 h, cells were pretreated with ERK and JUNK inhibitors (5 μM GW5074 and 0.5 μM U0126, respectively) for 1 h and then treated with TWEAK. TWEAK readily induces MMP-9 expression. Both inhibitors significantly inhibit TWEAK-induction of MMP-9 in the cell lysate (densitometric analysis of zymograms). (**E**) Exogenous recombinant human MMP-9 (rhMMP-9, 250 ng/ml for 24 h) enhanced the permeability (Pe) of the endothelial cell monolayer by 20%. (**F**) Inhibition of MMP activity with the broad-spectrum MMP inhibitor RXPO3 for 24 h has no effect on TWEAK- or TNFα-increased Pe and barrier impairment. In (A,B,D-F) * *P* < 0.05 according to Student’s *t* test.

Published reports indicate that TWEAK induces proteolytic activity, notably metalloproteinase activity, in different cell types [18,26]. We have also observed, in a transcriptomic analysis of hMEC/D3 cells treated for 24 h by TWEAK, that the mRNAs encoding several MMPs (MMP-12, MMP-17, MMP-28) were up-regulated, including MMP-9 (data not shown). We thus evaluated the effects of TWEAK on the gelatinolytic activity of hCMEC/D3 cells. We used *in situ* zymography following exposure to TWEAK (100 ng/ml) for 24 h. We found significantly increased gelatinolytic activity in TWEAK- or TNF-treated hMEC/D3 cells, compared with nontreated cells. In some cells, gelatinolytic activity appeared to delineate cells, suggesting localization at the plasma membrane (Figure 5A). Among proteinases that have gelatinolytic activity are the gelatinases MMP-2 and MMP-9. hCMEC/D3 cells were exposed to TWEAK and TNF for 24 h and gelatin-based gel zymography was performed on cell lysates and serum-free media conditioned by the treated and nontreated cells (Figure 5B). Nontreated cells showed constitutive expression of a 68 kDa gelatinase corresponding to the pro-form of MMP-2 and weaker expression of the 105 kDa gelatinase corresponding to the molecular weight of pro-MMP-9. Densitometric scanning of the zymograms indicated that MMP-2 levels (cellular and secreted forms) remained unchanged following TWEAK and TNF treatment. Secreted levels of MMP-9 also remained identical to control. In contrast, cellular MMP-9 pro-form levels, as well as active MMP-9, increased significantly following TWEAK and TNF treatment. To assess whether increased MMP-9 levels correlated with increased steady-state MMP-9 mRNA levels as suggested by the transcriptomics analysis, we used qPCR; an increase in MMP-9 mRNA expression was indeed observed in hCMEC/D3 cells 24 hours after treatment with TWEAK and TNF (Figure 5C). 

In fibroblast and skeletal muscle cells, TWEAK is reported to activate MAPKs and regulate the expression of MMP-9 [27,28]. To assess whether this is also the case in brain endothelial cells, cultures were incubated for 1 h before adding TWEAK in the presence GW5074 and U0126, which are respectively inhibitors for ERK1/2 and MEK2 involved in the MAPK signaling pathways. Densitometric scanning of zymograms indicates that MAPK inhibitors efficiently suppressed the up-regulation of MMP-9, whose expression remained at basal levels (Figure 5D) and had no effect on the expression or activity levels of MMP-2 (data not shown). These results suggest that in human brain endothelial cells, TWEAK induces MMP-9 up-regulation via the MAPK signaling pathways.

**Figure 6:**
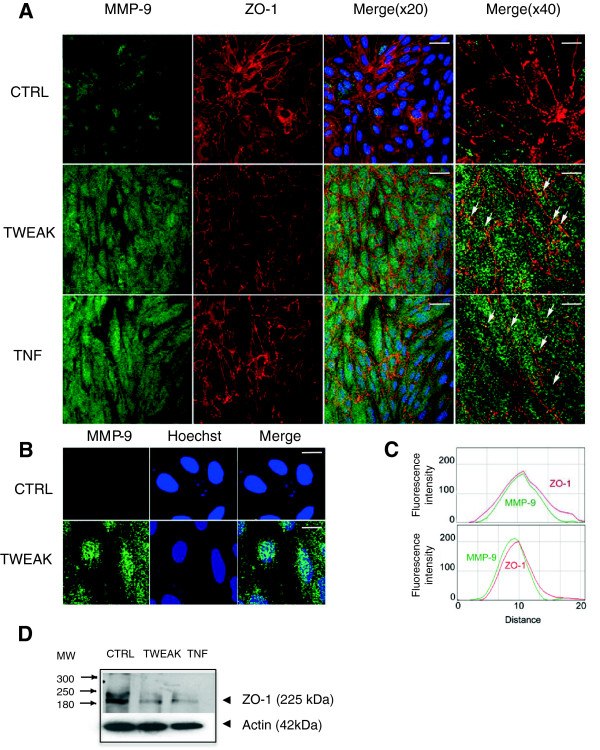
**MMP-9 and Zo-1 expression and distribution in hCMEC/D3 cells treated with TWEAK.** (**A**) Colabeling immunocytochemistry showing expression and distribution of MMP-9 and ZO-1 in control (CTRL), TWEAK-, and TNFα-treated hCMEC/D3 cells. MMP-9 showed a punctuate, vesicular-like pattern distributed throughout the cytoplasm that was increased in the TWEAK- and TNFα-treated cells. Superimposition of MMP-9 and ZO-1 images shows colocalization of both proteins (Merge (×20), scale bar 10 μm) in yellow-orange along the plasma membrane, visible as distinct puncta (arrows in Merge (×40), scale bar 2.5 μm). Note the global down-regulation of ZO-1 expression in TWEAK and TNF-treated hCMEC/D3 cells as compared with CTRL. (**B**) Confocal analysis of a single *z*-axis plan of the TWEAK-treated cells shows nuclear accumulation of MMP-9. Scale bar 2 μm. (**C**) Kymographs were constructed and analyzed with ImageJ software from single-pixel width lines taken from each channel of the confocal images in A (Merge (×40)). Profiles of the signal intensities of MMP-9 (green line) and ZO-1 (red line) measured along the single-pixel width lines drawn in Merge (×40) indicate colocalization. (**D**) Western blot analysis of protein extracts from TWEAK-treated hCMEC/D3 cells following separation by SDS PAGE and transfer onto nitrocellulose membranes, which were probed with primary antibodies against ZO-1 and actin. Both TWEAK and TNF treatments diminish ZO-1 expression in the hCMEC/D3 cells. Representative Western blot of three independent experiments.

We next evaluated the effects of exogenous recombinant human MMP-9 on the permeability of the hCMEC/D3 monolayer. The addition of 250 ng/ml rhMMP-9 for 24h enhanced the permeability of the endothelial cell monolayer by 20% (Figure 5E). Considering that TWEAK increases both Pe of the hCMEC/D3 monolayer and MMP-9 levels and that exogenously applied rhMMP-9 also increases Pe, we assessed whether TWEAK-increased Pe could be modulated by MMP inhibitors. TWEAK and the broadband MMP inhibitor RXPO3 were applied to the hCMEC/D3 monolayer for 24h and Pe was assessed. We find that inhibition of MMP activity neither prevents barrier impairment nor leads to a significant barrier recovery (Figure 6F).

To investigate the cellular distribution of MMP-9 in nontreated and TWEAK-treated endothelial cells, we used an antibody for MMP-9 whose specificity had been previously validated on neuroblastoma N2a cells transfected with MMP-9-GFP-constructs [25]. In nontreated and TWEAK-treated hCMEC/D3 cells, MMP-9 showed a punctuate, vesicular-like pattern distributed throughout the cytoplasm (Figure 6A). MMP-9 immunolabeling was clearly increased in the TWEAK- and TNF-treated cells. Detailed analysis of the labeled endothelial cells also indicates perinuclear accumulation of MMP-9, presumably in the Golgi and trans-Golgi network. We show that MMP-9 is localized in part at the membrane of brain endothelial cells. In agreement with our previous findings in other cell types of the CNS [25,29], MMP-9 was also localized in the nucleus of brain endothelial cells (Figure 6B).

### TWEAK down-regulated expression of ZO-1, a major component of HCMEC tight junctions

Because ZO-1 is exclusively located in tight junctions but also constitutes a substrate for MMP-9 [30], we assessed expression of this protein in the hCMEC/D3 cells under TWEAK exposure. Using double labeling experiments combining antibodies against MMP-9 and against ZO-1, we show that MMP-9 and ZO-1 can be colocalized at discrete areas of the plasma membrane (Figure 6A, 6C). Using Western blot analysis, we show that soluble TWEAK induced a down-regulation of the two isoforms, α+ and α−, of ZO-1 (Figure 6D).

## Discussion

TWEAK has been shown to induce various biological responses through binding to its receptor Fn14 [31], including angiogenesis, osteoclastogenesis, skeletal muscle wasting, and apoptosis [27,31]. TWEAK is also known as a proinflammatory cytokine involved in tissue injuries including brain inflammatory processes [10,17,22,32]. Disruption of the BBB occurs in a number of pathological conditions, including cerebral ischemia, head trauma, CNS infections, and MS [12,33-37], and results in the development of cerebral edema, which is a frequent cause of mortality in patients. Thus, understanding the pathophysiological processes leading to disruption of the barrier and increased permeability is crucial for the development of new therapeutic strategies. *In vivo* administration of TWEAK in mice has been shown to induce cerebrovascular permeability [12]. In this study, we assessed the effects of TWEAK at the molecular level on a human *in vitro* model of the BBB. We show for the first time that soluble TWEAK: (i) induced proliferation of human brain endothelial cells; (ii) promoted an inflammatory pattern of these cells, notably by stimulating secretion of cytokines, (iii) modulated the levels of cell adhesion molecules which is crucial for leukocyte-endothelium interaction and finally, (iv) modulated the expression of MMP-9 and ZO-1 and increased the permeability of the endothelial cell monolayer.

We and others have previously shown that TWEAK/Fn14 interaction induces proliferation of astrocytes and endothelial cells, production of proinflammatory cytokines and expression of adhesion molecules [7,8,11]. Nevertheless, there was a lack of data about the microvascular endothelial cerebral cells involved in the BBB, which represent a major cellular component of neuroinflammation. Data generated by the study of TWEAK on HUVECs, for example, cannot be directly applicable to interactions between white blood cells and endothelial cells at the BBB. We demonstrated in this study that immortalized or primary HCMECs respond to TWEAK/Fn14 interaction by adopting an inflammatory profile that is associated with an increased permeability of the monolayer formed by these cells in an *in vitro* BBB model. It is worth noting that the proliferative property of TWEAK on hCMEC/D3 is much more dramatic than that on cytokine induction. This observation could be explained by either a specific action of TWEAK or a synergistic effect of TWEAK combined with a TWEAK-enhanced bFGF endothelial cell proliferative effect. In fact, our proliferation culture medium is enriched in bFGF and it has been shown by others that TWEAK can act in concert with bFGF to regulate endothelial cell proliferation [7,8]. Our results suggest that soluble or membrane TWEAK expressed by blood monocytes or nervous tissue macrophages may regulate leukocyte recruitment across the BBB by promoting the production of the CCL-2 and IL-8 chemokines by HCMEC, but also by enhancing the expression of ICAM-1 at their cell surface, an adhesion molecule involved in leukocyte-endothelium interaction during transendothelial migration [38]. Interestingly, CCL2, known to be involved in neuroinflammatory processes, not only promotes leukocyte chemotaxis but also compromises the BBB [39]. In fact, CCL2 mediates redistribution of tight-junction proteins and reorganization of the actin cytoskeleton in brain endothelial cells [40-42], leading to altered BBB functions. We thus suggest that induced secretion of CCL2 by HCMECs may be one of the processes elicited by TWEAK to increase BBB permeability.

Our results suggest that TWEAK induced the expression of MMP-9 through the activation of MAPK signaling pathway in human cerebral endothelial cells. The ability of TWEAK to modulate MMP-9 expression was previously reported in a few studies including the work of Chicheportiche *et al*. in 2002 [43]. Li *et al*. [27] have also shown that TWEAK affects the expression of several genes of the MMP family in skeletal muscle. It was also proposed that TWEAK plays a role in MMP-3 and MMP-1 up-regulation [43,44]. Finally, TWEAK has been shown to enhance MMP-9 expression in several cell types, including macrophages [45], C2C12 myotubes [28], and mouse astrocytes [12]. Nevertheless, the expression of these MMPs and their potential role in structural and functional deterioration of BBB during pathological conditions remain largely unknown. Asahi *et al*. [15,30] demonstrated that MMP-9 has a direct effect on the permeability of the neurovascular unit. Interestingly, we show that cerebral endothelial cells under TWEAK treatment express more of the pro-MMP-9 than of the active MMP-9. This is in agreement with several studies involving gelatin zymography showing that it is essentially pro-MMP-9 that is induced in glial cells, neurons, and endothelial cells [16,29,46,47]. This observation may reflect a rapid and local activation of MMP-9 in these cells by sharp activation mechanisms. Our study provides the first evidence showing that in endothelial cells, it is only the cellular MMP-9, presumably including membrane bound-MMP-9, that is increased by TWEAK, while there is no induction of secreted MMP-9. We hypothesize that MMP-9 may play a local role in cytosolic and membrane proteolytic activity under physiological and pathological conditions. A number of *in vitro* results, including some from our laboratory, indicate that pro- and active forms of MMP-9 are also localized in the membrane, for example in neural cells [25,29]. Previous studies suggest that activation of MMP-9 pro-enzyme occurs by binding at the cell surface, and that the activated enzyme is then rapidly degraded, preventing excess activity [48]. Therefore, only very low levels of active MMP-9 may be present in cells at any given time. Further studies are required to identify potential MMP-9 activation events and elucidate their time course in more detail. We also reported MMP-9 localization in the nucleus of hCMEC/D3 and show increased nuclear levels following stimulation with TWEAK. While MMP-9-interacting proteins or targets in the cell nucleus are unknown, there is rationale for its interaction with Ku, a nuclear DNA repair protein, considering that both proteins have been shown to interact, notably at the cell surface [49].

Treatment of hCMEC/D3 with MEK and ERK inhibitors significantly reduced TWEAK-induced MMP-9 gelatinolytic activity, supporting the idea that MAPK pathways are involved. It has been shown that the binding of TWEAK to its receptor Fn14 could result in activation of the MAPK pathway *in vitro*[50,51]. Moreover, activation of MAPK pathways has been described in endothelial cells in several neuropathological processes, such as cerebral ischemia [52,53], head trauma [54], and seizures [55].

Our data support the contention that MMP-9 rather than MMP-2 contributes at least in part to the increased permeability of the HCMEC monolayers. However, we cannot exclude the possibility that other MMPs or proteinases from other families also contribute to BBB demise. Indeed, while we show that recombinant MMP-9 can increase the permeability of our BBB model, and while MMP inhibitors have proven beneficial effects in, for example, animal models of hypoxia ischemia [56], we also show that increased BBB permeability *in vitro* cannot be prevented with a broad-spectrum MMP inhibitor. One hypothesis is that active MMP-9 (or other MMPs) was not efficiently inhibited if present in the plasma membrane. Indeed, it has been shown that even high-affinity endogenous inhibitors for MMP-9, such as TIMP-1, cannot inhibit MMP-9 when present at the plasma membrane [57]. Proteinases may synergize with other molecular events to promote BBB demise, for example, CCR2-dependent CCL-2 biological effects described during neuroinflammation [40,58]. Tight-junction integrity is known to play a key role in brain homeostasis and a loss of tight-junction proteins is commonly observed in neuroinflammatory and neurodegenerative disorders. Tight-junction proteins, including ZO-1, are thought to have both structural and signaling roles and are linked to the actin skeleton of the endothelial cells. We show that treatment of HCMEC with soluble TWEAK resulted in decreased levels of ZO-1. In a previous study, an emphasis has been placed on the fact that ZO-1, albeit intracellular, is a substrate of MMP-9 [59] while *in vivo* studies underlined that this substrate was degraded by MMP-9 after ischemia [16]. Moreover, it was recently shown that suppression of MMP-9 expression in brain microvascular endothelial cells induced an increase of gene and protein expression of ZO-1 in these cells [60].

In summary, our study demonstrates that TWEAK modulates the expression levels of cytokines, CAMs, tight-junction proteins, and MMPs in cultured endothelial cells, and alters the permeability of an *in vitro* BBB model based on these cells. These results are in agreement with our previous results demonstrating *in vivo* a role for TWEAK in regulation of immune cell infiltrates in the CNS during experimental autoimmune encephalomyelitis (EAE) [10].

We propose a model in which soluble TWEAK is released during neuro-inflammation, or membrane TWEAK is presented by monocytes, and binds to Fn14 receptors on CNS endothelial cells, resulting in secretion of proinflammatory and chemoattractant cytokines, expression of cell adhesion molecules, activation of the MAPK pathway, and induction of MMP-9, with a resulting disruption of the tight-junction structure and increase in BBB permeability and diapedesis. Our studies predict that it may be beneficial to block the TWEAK/Fn14 pathway as a therapeutic modality in BBB breakdown.

## Abbreviations

BBB: Blood brain barrier; bFGF: Basic fibroblast growth factor; BrdU: Bromodeoxyuridine; CNS: Central nervous system; EAE: Experimental autoimmune encephalomyelitis; ELISA: Enzyme-linked immunosorbent assay; FACS: Fluorescence-activated cell sorting; FCS: Fetal calf serum; FGF: Fibroblast growth factor; Fn14: Fibroblast growth factor-inducible 14; HCMEC: Human cerebral microvascular endothelial cells; HMEC: Human microvascular endothelial cell; HUVEC: Human umbilical vein endothelial cell; kDa: Kilodalton; LY: Lucifer yellow; MAPK: Mitogen-activated protein kinase; MMP: Matrix metalloproteinase; PBS: Phosphate-buffered saline; PCR: Polymerase chain reaction; Pe: Permeability coefficient; PFA: Paraformaldehyde; qPCR: Real-time PCR; RH: Ringer-HEPES; RT: Reverse transcriptase; SDS PAGE: Sodium dodecyl sulphate polyacrylamide gel electrophoresis; TNF: Tumor necrosis factor; TEER: Transepithelial electric resistance; TWEAK: Tumor necrosis factor-like weak inducer of apoptosis; ZO-1: Zonula occludens-1.

## Competing interests

The authors declare that they have no competing interests.

## Authors’ contributions

DS, OS, and JW performed the experiments. CP and POC participated in the design of the study. CP helped to draft the manuscript. SDJ and MK conceived the study and drafted the manuscript. SDJ coordinated the study. All authors have read and approved the final version of the manuscript.
